# Cachexia as Evidence of the Mechanisms of Resistance and Tolerance during the Evolution of Cancer Disease

**DOI:** 10.3390/ijms22062890

**Published:** 2021-03-12

**Authors:** Antonio Maccio, Elisabetta Sanna, Manuela Neri, Sara Oppi, Clelia Madeddu

**Affiliations:** 1 Department of Gynecologic Oncology, Businco Hospital, ARNAS G. Brotzu, 09121 Cagliari, Italy; dr.elisabettasanna@gmail.com (E.S.); manu.neri11@hotmail.it (M.N.); 2 Hematology and Transplant Center, Businco Hospital, ARNAS G. Brotzu, 09121 Cagliari, Italy; sara.oppi@aob.it; 3 Department of Medical Sciences and Public Health, University of Cagliari, 09124 Cagliari, Italy; clelia_md@yahoo.it

**Keywords:** cancer cachexia, resistance, tolerance, inflammation, muscle wasting, interleukin-6

## Abstract

During its evolution, cancer induces changes in patients’ energy metabolism that strongly affect the overall clinical state and are responsible for cancer-related cachexia syndrome. To better understand the mechanisms underlying cachexia and its metabolic derangements, research efforts should focus on the events that are driven by the immune system activation during the evolution of neoplastic disease and on the phenomena of “resistance” and “tolerance” typically involved in the human body response against stress, pathogens, or cancer. Indeed, in the case where resistance is not able to eliminate the cancer, tolerance mechanisms can utilize the symptoms of cachexia (anemia, anorexia, and fatigue) to counteract unregulated cancer growth. These notions are also sustained by the evidence that cancer cachexia may be reversible if the resistance and tolerance phases are supported by appropriate antineoplastic treatments. Accordingly, there is no doubt that anticachectic therapies have an irreplaceable role in cases of reversible cancer cachexia where, if harmoniously associated with effective antineoplastic therapies, they can contribute to preserve the quality of life and improve prognosis. Such anticachectic treatments should be based on targeting the complex immunological, inflammatory, and metabolic pathways involved in the complex pathogenesis of cachexia. Meanwhile, the role of the anticachectic therapies is very different in the stage of irreversible cachexia when the available antineoplastic treatments are not able to control the disease and the resistance mechanisms fail with the prevalence of the tolerance phenomena. At this stage, they can be useful only to improve the quality of life, allowing the patient and their family to get a better awareness of the final phases of life, thereby opening to the best spiritual remodulation of the final event, death.

## 1. Introduction

During its evolution, cancer induces changes in patients’ energy metabolism that strongly affect the overall clinical state [1]. A plethora of symptoms that involve various organs and systems are linked with cancer-associated metabolic changes, including anorexia, nausea, weight loss with reductions in lean mass and adipose tissue, and increased energy metabolism with changes in glucose, lipid, and protein metabolism. These changes are often responsible for cancer-related cachexia syndrome—a condition associated with malignancy identified by involuntary weight loss accompanied by chronic inflammation, fatigue, anorexia, and anemia [2]. This syndrome affects cancer patients with metastatic disease with a variable incidence among different tumor types and is responsible for about half of all cancer deaths worldwide [3], although it may be reversible in some phases of the neoplastic disease. A consensus agreement defined cancer cachexia as a multifactorial syndrome characterized by the ongoing loss of skeletal muscle mass (with or without the loss of fat mass) that cannot be fully reversed by conventional nutritional support, and thus, ultimately leads to progressive functional impairment [4]. The consensus diagnostic criteria for cachexia are ≥5% weight loss, or ≥2% weight loss in individuals already showing decreases in body weight and height (body mass index (BMI) < 20 kg/m^2^) or skeletal muscle mass (sarcopenia) [4]. Researchers agree that cancer cachexia syndrome can develop progressively through various stages, from pre-cachexia to cachexia and refractory cachexia. Cachexia severity can be classified according to the degree of depletion of energy stores and protein (based on BMI) in combination with the degree of ongoing weight loss [4]. Assessments to guide disease classification and clinical management should include the following domains: anorexia or reduced food intake, catabolic drive, i.e., inflammation and tumor growth, muscle mass and strength, and functional or psychosocial impairment.

Despite the evidence presented above, early, and accurate diagnosis is lacking in clinical practice and a standardized effective treatment has not yet been established. Meanwhile, based on the relevant clinical impact of this syndrome, a therapeutic approach based on the depth of knowledge of its complex pathogenesis must be implemented.

In this review, we aim to discuss the main changes in metabolism that define cachexia and the key mechanisms and mediators contributing to them. We focus on the role that resistance and tolerance, involved the host’s response against a pathogen (i.e., cancer cells), have in determining the cachexia syndrome during the evolution of neoplastic disease. Additionally, we provide the rationale for a potential therapeutic approach targeting these pathways.

## 2. The Pathophysiology of Cancer Cachexia

Cancer cachexia pathogenesis is characterized by a negative protein and energy balance driven by a combination of abnormal energy utilization and reduced food intake. Both the tumor growth and the chronic immune activation that attempts to halt it can elicit an overall metabolic state that is characterized by the following [1]:

- Increased resting energy expenditure with progressive weight loss.
- Increased requirements for glucose and other energy substrates.
- Reduced food intake consequent to anorexia, nausea, and vomiting.
- Increased gluconeogenesis, which leads to protein and lipid depletion and weight loss.
- Compromised glucose use because of hypoinsulinemia and peripheral insulin resistance.
- Inadequate detoxification systems.
- Oxidative stress with elevated reactive oxygen species (ROS)-induced oxidative damage to DNA, membrane lipoproteins, enzymes, and coenzymes.
- Chronic inflammation.
- Anemia.
- Immunodepression.

It is assumed that these changes are caused by the interactions between the immune system and the tumor, although it is difficult to establish the exact moments that define the onset of these changes [5,6]. Considering that we often encounter patients that are already cachectic at the time of diagnosis, this difficulty may hinder the ability to prevent, prognosticate, and treat this syndrome in a timely and effective manner.

To better understand the mechanisms underlying cachexia, research efforts should focus on the events that are driven by the immune system activation during the evolution of neoplastic disease. In particular, we should focus on the phenomena of resistance and tolerance typical of the human body’s response to stress, pathogens, or cancer [7,8].

Only then, might we be able to better understand the mechanisms involved in cachexia, determine its complex clinical picture, and implement the new knowledge to identify it early, define its clinical components properly, and develop targeted and effective therapeutic interventions.

### 2.1. Resistance and Tolerance Mechanisms Underlie Cancer Cachexia

**Figure 1:**
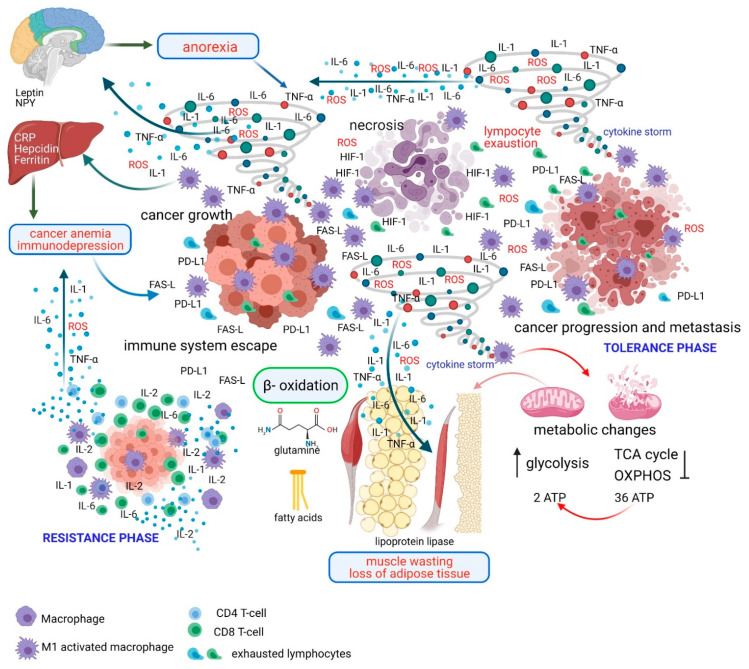
Mechanism of resistance and tolerance and pathogenesis of cachexia during the evolution of cancer. In the resistance phase the immune system recognize the antigenic diversity of the tumor and tries to eliminate it. Macrophages, dendritic cells and T and B cells are the main participants. In this phase the presence of cancer cells and the activation of the immune system with the release of proinflammatory cytokines (IL-6, IL-1, TNF-α), HIF-1, and ROS determine specific changes in energy metabolism (increased glycolysis and impaired TCA cycle and OXPHOS) that promote uncontrolled tumor growth. The tumor growth overcoming the resistance phase underlines the lack of efficacy of the specific immune response (lymphocyte exhaustion) followed by the mac-rophage-mediated inflammatory response (cytokine storm) whose persistence leads to the phenomena of tolerance and related symptoms (anorexia, anemia, muscle wasting, loss of adipose tissue). Cachexia as an expression of tolerance aims to minimize the damages induced both by the cancer-growth and by immunopathology. Abbreviations: NPY, Neuropeptide Y; CRP, C-reactive Protein; IL, Interleukin; TNF, Tumor Necrosis Factor; PD-L1, Programmed death-ligand 1; ROS, reactive oxygen species; HIF, hypoxia-inducible factor; FAS-L, Fas ligand; TCA, tricarboxylic acid; OXPHOS, Oxidative phosphorylation; ATP, Adenosine Triphosphate. Figure created with BioRender.com (accessed on 12 March 2021).

Cancer cells can be considered pathogens; therefore, their related pathology depends on the extent of their growth and the effectiveness of the immune system response. Indeed, a tumor has always been considered an invader to the body in which it grows, even if it appears, grows, and develops in its native environment. The body’s response to an invading pathogen can be divided into two phases, whose mechanisms define the body’s defensive capacity. The initial phase is called resistance, where the body tries to get rid of the pathogen, and the second is called tolerance, where the body attempts to limit the health impact caused by the presence of the pathogen [9]. Resistance reduces the cancer cell burden once oncogenesis is initiated [7]. In the resistance phase, the immune system recognizes and tries to eliminate the antigenic diversity of the tumor. The clinical manifestation of cancer highlights the poor immunogenicity of tumors and the reduced effectiveness and inability of the immune system to control tumor cell growth due to escape mechanisms utilized by the cancer. Macrophages, dendritic cells (DCs), and T and B cells are the main participants in antitumor resistance. Yet, in this phase, the combination of the activation of the immune system, with related proinflammatory cytokines, and the presence of neoplastic cells, dictate specific changes in cellular energy metabolism that support uncontrolled cancer growth (Figure 1).

The phase of resistance against cancer is characterized by (i) the uncontrolled growth of the cancer cell, through progression, invasion, and metastasis, which goes toward the death of the host leading to the destruction of the cancer itself. (ii) The activation of the immune system, known as immunosurveillance, follows the classical steps of antigen recognition, antigen presentation to effector and regulatory T cells, and the physiological end of the immune response by activation of the immune checkpoint pathways. 

Therefore, the growth of a tumor that overcomes the mechanisms of resistance underlines a lack of efficacy of the specific immune response, which is followed by a macrophage-mediated chronic inflammatory response with related symptoms, whose persistence leads to the phenomena of tolerance (Figure 1). This phase occurs to limit the damages related to the chronic activation of the immune system.

Tolerance is a defense strategy that reduces the negative impact resulting from the interaction between the tumor and the patient [7]. Although the resistance strategy might be crucial for protecting the patient, it also carries a significant cost to the fitness of the patient [7], as the destruction and elimination of cancer cells is accompanied by collateral tissue damage. Collectively, the negative impact of immune defenses on the fitness of the patient is referred to as immunopathology [10], which is considered an unavoidable consequence of this process. In general, the degree of immunopathology is positively correlated with the magnitude and duration of the immune response. Thus, the optimal immune response is determined by the balance between the efficient clearance of cancer and an acceptable level of immunopathology [11].

The tolerance strategy is activated because the damage related to inflammation has a cost as lethal to the patient as the damage induced by the cancer. Unlike resistance mechanisms, tolerance does not directly affect the pathogen burden. Rather, tolerance decreases the susceptibility of the patient to damage of tissues or other fitness costs caused by the cancer or by the immune response against it. In summary, the patient sustains two types of tissue damage: (i) direct damage by the neoplasm and (ii) immunopathological damage. Accordingly, the patient’s body might utilize two types of tolerance mechanisms, one minimizing the cancer growth-induced damage, and the other minimizing the immunopathological-induced damage. Both the cancer growth and the immune response can cause damage to tissues by disrupting the normal architecture of tissues, homeostasis, and function [7].

In principle, almost any physiological process can be negatively affected by either the growth of cancer or the immune and inflammatory responses they elicit. The mechanisms that normally maintain the homeostasis of various physiological systems are likely to contribute to the tolerance of the patient to cancer. Pathological outcomes associated with cancer growth might arise when the degree of tissue damage or the disruption of the patient’s physiology exceeds the capacity of tolerance mechanisms.

Similarly, most infections in animals and humans lead to dramatic changes in behavior, resulting in fatigue, anorexia, social withdrawal, fever, and sleep alterations, which are collectively known as sickness behaviors [12]. Although sickness behavior is assumed to be adaptive, it is not clear whether and how it helps the infected patient. For example, fatigue is thought to preserve the energy to better fight the infection, and it is also commonly accompanied by anorexia, and therefore, less energy intake and reduced energy consumption.

Loss of appetite (i.e., anorexia) is a common behavior exhibited by sick animals facing an immune challenge [13]. Traditionally, anorexia was considered an adverse secondary response to infection that served no function to the host; immune responses are energetically expensive, and thus, an infection-induced reduction in food intake seems paradoxical [14]. However, since this phenomenon occurs in so many animals, an alternative explanation is that this response might be a conserved adaptive strategy to increase the chances of surviving an infection [14]. Experimental evidence from anorexia and acute starvation studies is consistent with this notion and suggests that this behavioral change might be advantageous and actively induced by the host during infection [15]. Indeed, anorexia is characterized by a reduction of food intake, possibly aimed at limiting nutrient availability to invading pathogens [16]. Glucose dietary uptake and its endogenous synthesis are tightly regulated to maintain blood glucose levels within a homeostatic range [17]. Enforcing this homeostatic range is particularly challenging during an infection, given that the pathogens and host compete for glucose [18]. It is plausible that the infected host evolved strategies, including anorexia, to limit glucose availability to pathogens while maintaining glucose levels within a range compatible with survival [13,19]. While protective against some classes of pathogens, anorexia carries a high evolutionary trade-off since nutrient deprivation can compromise host homeostasis [20]. Such behavior likely also occurs in patients with cancer.

Therefore, although sickness behavior might have some undefined positive effects on resistance, its benefits might be largely related to promoting the tolerance of the patient to cancer. As such, anorexia, and fatigue, for example, might help preserve vital processes and promote stress tolerance in multiple tissues. Therefore, the cancer cachexia syndrome could be the last form of resistance against an enemy (i.e., cancer).

Therefore, just by analyzing the mechanisms of “resistance” and “tolerance”, we can attempt to improve our understanding of the cancer cachexia syndrome. In the case where resistance is not able to eliminate the cancer, tolerance mechanisms can utilize the symptoms of cachexia (anemia, anorexia, and fatigue) to counteract unregulated cancer growth (Figure 1).

**Figure 2:**
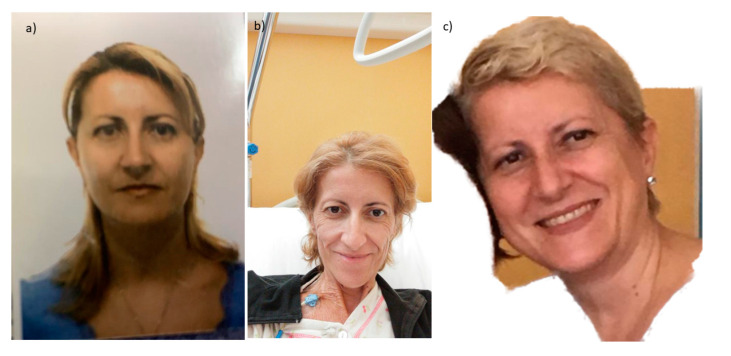
Reversibility of cancer cachexia. The images report the case of a female patient with advanced ovarian cancer, which exemplify the change of clinical objective status of a patient before cancer diagnosis (**a**), at cancer diagnosis with features of cancer cachexia (**b**), and after surgical and medical treatment that obtained a complete response with clinical resolution of cancer cachexia (**c**). The patient gave signed informed consent for the publication of the images.

The above-presented evidence is sustained by a theme that has received little attention thus far, that cancer cachexia may be reversible. In fact, in clinical practice, several patients exhibiting symptoms of cachexia (weight loss ≥ 5–10 % of ideal weight in the last 3–6 months) exhibit a significant resolution of the phenomena and associated symptoms with the reduction of tumor burden. Once a patient, even in an advanced stage of the disease, achieves a clinical response, especially a complete response, they might regain their appetite with a resolution of anorexia, gain weight with the improvement of lean body mass, and become free of other symptoms associated to cachexia, such as anemia and immunodepression (Figure 2). In other words, cachexia is reversible if the resistance and tolerance phases are supported by appropriate treatments, as during a bacterial infection with the use of antibiotics.

The irreversible form of cachexia that leads to death develops when the available therapies are not able to control the disease and the resistance mechanisms fail with the prevalence of the tolerance phenomena.

### 2.2. Role of Hypoxia-Inducible Factors (HIFS) in Metabolic Changes of Cachexia

Immunopathology leading to the establishment of cachexia is characterized by necrosis with associated hypoxia and consequent synthesis of adaptation factors, mainly the hypoxia-inducible factor (HIF). We believe that the production of HIF may be the key event to drive the metabolic changes in the cancer cachexia syndrome [21,22].

HIFs belong to a family of oxygen-regulated transcription factors that coordinate the transcriptional response to hypoxia. They sense hypoxia and induce a variety of transcriptional, metabolic, and morphological responses to maintain cellular homeostasis. When oxygen is limited, HIF-α is stabilized and translocated to the nucleus, where it binds to conserved hypoxia response elements (HREs) in the promoter regions of HIF-regulated genes. Thus, HIF-α transcriptional activity is regulated in an oxygen-dependent manner by controlling protein stability in the cytosol and transcriptional coactivator binding in the nucleus. Such regulation is mediated by the hydroxylation of specific amino acids by oxygen-dependent dioxygenases, namely, the proline-targeting prolyl hydroxylase domain (PHD) enzymes 1, 2, and 3, and the asparagine-targeting factor inhibiting HIF (FIH) enzyme. Both classes of enzymes catalyze the oxidative decarboxylation of 2-oxoglutarate (2-OG, or α-ketoglutarate (α-KG)), which produces carbon dioxide and succinate as byproducts. These enzymes require iron in the ferrous (Fe^2+^) oxidation state, which is maintained by the reducing action of ascorbic acid [21]. Under hypoxic conditions, the hydroxylase activity of PHDs and FIH is inhibited, blocking the binding and ubiquitination of HIF-α by the von Hippel-Lindau (pVHL) tumor suppressor, leading to the cytoplasmic stabilization of HIF-α subunits [23]. Then, the accumulated HIF-α translocate to the nucleus, where it binds to HREs, dimerizes with the HIF-β subunits, and recruits additional transcriptional coactivators to transactivate the transcription of hypoxia-responsive genes [24].

In cancer, HIF is produced to ensure efficient energy metabolism as the growing tumor experiences low oxygenation. Under these conditions, HIF-regulated gene expression diverts glucose and fatty acid-derived carbons from being catabolized to acetyl-CoA, while glutamine-derived carbons are diverted from being catabolized to succinyl-CoA. The overall result is increased glycolysis with a halt of mitochondrial oxidative phosphorylation (OXPHOS). Because glycolytic ATP production is less efficient (2 ATP/glucose) than OXPHOS-mediated ATP production (36 ATP/glucose), HIF also upregulates plasma membrane glucose transporters (GLUT1 and GLUT3) and glycolytic enzymes such as hexokinase 2 (HK2), aldolase A (ALDA), and enolase 1 (ENO1) to increase glycolytic flux and maintain ATP homeostasis [21]. Based on these functions, HIF production belongs to the typical hallmarks of cancer, which include the Warburg effect—the metabolic shift from the tricarboxylic acid pathway to glycolysis. Indeed, hypoxia is a prominent component of solid tumors, primarily as a pathophysiological consequence of disrupted microcirculation due to insufficient vascularization following rapid tumor growth. Beyond hypoxia, a switch to glycolysis has been described in tumors even under conditions of normoxia [22]. Through this metabolic switch, cancer cells escape the mechanisms of cell death induced by ROS and excess ATP produced through OXPHOS.

Notably, in several tumors, HIF-1 also stimulates the utilization of glutamine as alternative energy substrates for tumor growth [25].

Hypoxia also induces metabolic changes in immune cells, which are metabolically inert while quiescent and require notable reprogramming upon activation to provide sufficient ATP for performing their effector functions. The HIF pathway acts as a switch for metabolic phenotypes and is therefore a critical transcriptional regulator of immunity [22]. Aerobic glycolysis and HIF1α signaling are key features of activated immune cells, of which, activated and M1 polarized macrophages, are the best examples [26]. In these cells, the HIF pathway enables a metabolic switch that allows an appropriate response to the significant changes in energy requirements that occur upon activation and adaptation to the hypoxic conditions that prevail in inflamed tissue [27]. Foci of inflammation, generated under various conditions such as cancer growth, tissue insult, infection, and autoimmune responses, can form a difficult microenvironment for an immune cell. Hypoxia, acidosis, redox stress, and hypoglycemia are common features that cells must adapt to survive. Like the activated immune cells with high energy requirements (such as monocytes, macrophages, DCs, and T cells), cancer cells utilize a Warburg-like metabolism linked to increased glucose consumption and glycolytic flux [28]. Thus, the metabolic changes characteristic of cancer cells mirrors those observed in activated immune cells [29]. Thus, HIF-related pathways are linked to the physiological activation of the immune system, whereas they represent a pathological event in cancer cells.

The migration of immune cells to the tumor site might completely modify their relationship with oxygen and their metabolic profiles must change to accommodate changes in oxygen levels. Therefore, immune cells must change their metabolism as a function of the events associated with mobilization towards the “enemy” and to their activation state. Importantly, the metabolic changes mediated by HIF and used by neoplastic cells are not unique but rather inherent to the organism and are typically harnessed by activated immune cells [22].

HIF determines the metabolic shift in macrophages, which consequently become M1-polarized macrophages and produce proinflammatory cytokines typical of the patient with cancer cachexia. Through these cytokines, M1 macrophages sustain a state of chronic inflammation [30]. In summary, from the initial contact between cancer cells and immune cells in the tumor microenvironment, the presence of mediators of chronic inflammation transforms this condition into a systemic disease characterized by specific symptoms associated with tissue damage (immunopathology).

## 3. Changes in Energy Metabolism in Cachexia

Changes in energy metabolism, including increased resting energy expenditure associated with specific abnormalities in glucose, lipid, and protein metabolism are common features in advanced cancer patients, especially those who are cachectic [31]. Indeed, energy metabolism is involved in tumor growth and the substantial fuel consumption by the activated immune system [32]. Immune activation requires high energy consumption and is associated with a 25–30% increase in the basal metabolic rate, i.e., 1750–2080 kJ/day [33]. Thus, tumor development and its continuous growth led to an increase in resting energy expenditure, related to tumor mass and degree of aerobic versus anaerobic energy metabolism [34], accompanied by chronic activation of the immune system in an attempt to exert its anticancer response. This energy burden creates a metabolic scenario whereby two systems requiring a continuous supply of energy substrates, particularly glucose, are activated in the patient.

In differentiated tissues and under normoxic conditions, glucose is completely oxidized to CO_2_ and H_2_O through OXPHOS [35]. As described earlier, tumors mainly grow under hypoxic conditions under which the pyruvate dehydrogenase (PDH) complex is inactivated leading to the accumulation of pyruvate that is converted into lactate (anaerobic glycolysis) by lactate dehydrogenase (LDH). HIF is the key factor responsible for these metabolic changes [36].

Even in the presence of normal oxygen tension, cancer cells prefer aerobic glycolysis [37]. In tumor cells, this metabolic switch occurs through the upregulation of pyruvate dehydrogenase kinase, which regulates the final and rate-limiting step of glycolysis by inhibiting PDH activity and glucose flux in the Krebs cycle. Thus, cancer cells preferentially use glycolysis with enhanced lactate production rather than OXPHOS for glucose metabolism to support cell growth [38]. The net result is the conversion of glucose into lactic acid by an energy-inefficient process, which means that tumor growth requires more glucose than it would if mitochondrial respiration were fully utilized. This peculiar metabolic behavior is observed in activated immune cells in the tumor microenvironment, as demonstrated by Warburg [39].

Therefore, it seems that most metabolic abnormalities in cancer patients are precipitated by the use of glucose as the primary energy source by both the tumor and the activated immune cells [31,40]. Moreover, in cachectic cancer patients, notable alterations occur induced by the action of proinflammatory cytokines in the carbohydrate metabolism, including increased gluconeogenesis and Cori cycle, insulin resistance, and decreased glucose tolerance [41], all of which impair the utilization of glucose for energy. To meet the increased demand for glucose, the lactate passes from the tumor to the liver, where it is converted to glucose via a futile cycle, known as the Cori cycle, that requires 6 mol of ATP to generate 1 mol of glucose from 2 mol of lactic acid. This is a high energy-consuming process and may account for the additional energy expenditure in cancer patients of 300 kcal/day. Indeed, the Cori cycle is significantly increased in advanced cancer patients compared with healthy individuals [42].

The increased energy expenditure associated with the activation of these energy pathways contributes considerably to weight loss. Increased Cori cycle activity associated with increased lactate production has been observed in patients with progressive weight loss, whilst patients with no weight loss show normal levels of Cori cycle activity [43]. The compensatory increase in gluconeogenesis is associated with reduced synthesis of insulin and insulin resistance, in both the adipose tissue and skeletal muscles [44].

These changes in energy metabolism are exacerbated by the typical symptoms presented in advanced cancer patients, such as anorexia, nausea, and vomiting, which prevent normal nutrition and the regular supply of energy [45]. These symptoms, together with fatigue and catabolism of lean body tissues, belong to the sickness behavior elicited by the action of proinflammatory mediators on the central nervous system, which recognizes cytokines as the main molecular mediators [46]. All these changes contribute to creating a condition of severe energetic stress.

Indeed, altered cancer cell metabolism, associated with symptoms such as anorexia, requires increased energy substrates different from those deriving from diet to support tumor growth. Such substrates come mainly from increased catabolism of specific tissues typical of cachexia, such as adipose tissue and, in an even more severe way, skeletal muscle. This is because of the action of proinflammatory cytokines, mainly interleukin (IL)-6, that suppresses lipoprotein lipase and induces lipolysis and proteolysis with the release of fatty acids and other amino acids, including glutamine. It is well known that in high catabolic conditions, as cancer cachexia, the skeletal muscle is one of the most important sites of the release of glutamine, which, in turn, serves as one primary catabolic substrate of proliferative metabolism of cancer cells [47]. The altered energy metabolism in cancer cells is also characterized by high utilization of glutamine, through glutaminolysis and reverse tricarboxylic acid (TCA) cycle, mainly for the production of NAPDH2 and alanine that are used for fatty and nucleic acid synthesis and for the restoration of the molecules of TCA cycle (which is inhibited as a consequence of the decreased activity of the PDH complex) for different anabolic purposes of the intensively dividing cancer cells [48]. Both HIF, as well as other oncogenes (i.e., Myc), specifically potentiate the expression of glutaminase 1 (a key enzyme involved in glutaminolysis) and promote the utilization of glutamine carbon for cell growth [49].

Moreover, free fatty acids (FFA), deriving from altered lipid metabolism typical of cachexia, can be reused via β-oxidation in cancer cell mitochondria as an additional energy source in tumors [50]. Indeed, in cachexia proinflammatory cytokines (mainly TNF-α and IL-6) directly influence adipocyte metabolism by decreasing the activity of lipoprotein lipase (LPL), an enzyme that regulates the uptake of circulating triglycerides into adipocytes. Lipid metabolism is a complex sequence of events that determine whether the triglyceride pool within the adipocyte increases due to the processes of FFA uptake and lipogenesis or decreases due to the process of lipolysis. Circulating lipoproteins and triglycerides are first converted into FFA by the action of LPL, which is secreted by the adipocyte. FFAs can then enter the adipocyte via a fatty acid transporter and once inside the adipocyte, are converted into the triglyceride by a multi-step regulated enzymatic reaction involving acyl-CoA synthetase. Besides their activity on LPL, proinflammatory cytokines are also able to reduce the expression of FFA transporters in adipose tissue, thereby hindering the synthesis and entry of FFA into the adipocyte, and promoting lipolysis, leading to a concomitant increase in circulating FFA concentration [51]. Simultaneously, some tumor cells showed increased FFA uptake and utilization via β-oxidation. Indeed, it has been shown that cancer cells use fatty acids oxidation to oxidize FFA and derive energy by mitochondria but also to sustain their endogenous synthesis of lipids required by proliferating cells being components of cell membranes [52].

Thus, although cachexia is an attempt to preserve the patient body as a mechanism of tolerance, it may have also an advantage for the tumor. Both ongoing muscle wasting, as well as the loss of adipose tissue with specific changes of lipid metabolism, may serve as a source of energy and molecules to promote tumor growth by acting as high-energy onco-metabolites. Taken together, these findings suggest that cachexia may, at least in part, favors the growth and survival of a tumor that behaves as a “parasitic organism" [53].

### 3.1. Energetic Stress Response in Cancer Cachexia and Muscle Wasting

The decreased glucose availability resulting from metabolic changes associated with energetic stress induced by cancer development has been associated with an increase in the expression of stress response genes, including phosphoinositide 3-kinase/Akt/mammalian target of rapamycin (PI3K/Akt/mTOR), mitogen-activated protein kinases, p53, and components of inflammatory cytokine signaling pathways [54]. In advanced cancer patients, the muscle has decreased anabolic ability, as indicated by disturbances in muscle protein turnover in response to changes in feeding [55]. The PI3K/Akt/mTOR axis plays key roles in this process as a signaling pathway that regulates cell metabolism, energy balance, and proliferation [56]. The PI3K pathway is pivotal in cell cycle progression. Differentiated mammalian cells require mitogenic signals to exit quiescence (G0), re-enter the cell cycle, and move past the checkpoint in late G1. Cell cycle progression is driven by cyclin-dependent kinases activated by specific growth factors. The PI3K pathway is triggered during the G1/S phase transition and is associated with progression through the checkpoint; such pathway activation coincides with G1 events that lead to cyclin E/Cdk2 activation [57].

#### 3.1.1. Akt-Mediated Control of Metabolic Pathways

Akt is an important downstream effector of the PI3K pathway and participates in many related biological processes. In the absence of growth factor stimulation, all three Akt isoforms are nonfunctional in resting cells. Upon growth factor stimulation, Akt is activated in a PI3K-dependent process that involves receptor tyrosine kinases. Activated Akt regulates crucial molecular pathways, including cell survival, proliferation, apoptosis, and the response to insulin and nutrients [58]. Akt releases cells from blockade at both G1 and G2/M to increase cellular proliferation, and stimulates mTOR by phosphorylation at Ser2448, resulting in the activation of mRNA translation. Furthermore, Akt activates the inhibitor IκB kinase (IKK) through phosphorylation at Thr23, and thus, prevents the subsequent activation of the transcription factor nuclear factor (NF)-κB, which regulates the transcription of various pro-survival genes [59].

#### 3.1.2. mTOR-Mediated Control of Metabolic Pathways

mTOR, which belongs to the class of PI3K-related kinases, is another central regulator of cell growth and proliferation. mTOR is present in two complexes: mTOR complex (mTORC) 1 comprising mTOR raptor, a 40 kDa proline-rich Akt substrate (PRAS40), and mLST8; and mTORC2 comprising mTOR, rictor, mSin1, proctor, and mLST8. While mTORC1 primarily regulates protein translation, cell size, and proliferation by phosphorylating two key regulators of protein synthesis (S6K1 and eIF-4E-BP1), mTORC2 controls cell survival by directly phosphorylating, thereby activating Akt and serum and glucocorticoid-dependent kinase 1 [60]. Thus, mTORC1 regulates numerous metabolic pathways by sensing nutrient and growth factor levels [61]. Notably, mTORC1 plays a crucial role in restraining muscle protein synthesis, and its regulation by insulin/insulin growth factor (IGF)-1 signaling has been investigated extensively [62]. mTORC1 provides important negative feedback through the inhibition of PI3K at many levels. Additionally, mTORC1 can downregulate Akt by modulating the expression of insulin receptor substrate 1/2 via S6K to block PI3K [63].

During phases of energetic stress (such as those occurring in cachectic cancer patients), when glucose deprivation and hypoxia lead to decreased glycolytic flux, mTORC1 is inhibited by lowering ATP levels. This reduced ATP production and subsequent increase in the AMP/ATP ratio activates AMP-activated protein kinase (AMPK) [64], a heterotrimeric kinase composed of one catalytic subunit (α) and two regulatory subunits (β and γ) that acts as a cellular energy sensor and signal transducer in response to various metabolic pathways [65]. AMPK directly phosphorylates the tuberous sclerosis complex (TSC) 2, which activates this protein through an unknown mechanism, and suppresses mTORC1. Notably, these events are vital for cell survival under glucose deprivation conditions, although they inhibit protein synthesis in skeletal muscle [66].

Energy depletion can also inhibit mTORC1 independently of TSC2, in part by the direct phosphorylation of two serine residues on raptor by AMPK. These findings indicate that inhibiting mTORC1 is necessary for the cell-cycle blockade in response to energy stress [56]. This evidence further proves that symptoms such as anorexia leading to mTOR inhibition may represent a defense attempt (at the tolerance phase) initiated by the host to block tumor growth.

To date, mTOR blockade seems to be the main etiopathogenetic mechanism of protein synthesis failure in skeletal muscle typical of cachectic patients. Notable, in advanced cancer patients, specific conditions affecting energetic efficiency, including insulin-resistance, anorexia, and anemia, are capable of inhibiting mTOR. Therefore, pathways initiate that lead to muscle wasting during the evolution of neoplastic disease.

A recent clinical study in patients with lung cancer at the progressive stage of cachexia found that despite a gradual increase in Akt phosphorylation in muscle biopsies, a subsequent increase in the phosphorylation of downstream substrates (e.g., mTOR) was not observed [67]. These data suggest that muscle wasting in advanced cancer patients with cachexia is characterized by impaired Akt activity and suppressed mTOR signaling. Accordingly, this finding implies resistance to anabolic stimuli, which can have important consequences on muscle mass regulation given that mTOR is the central regulator of protein turnover [67].

In addition to these mechanisms of inhibiting mTORC1, low oxygen levels or hypoxia may inhibit mTORC1 signaling through multiple pathways. Hypoxia decreases cellular ATP levels by inhibiting metabolic pathways such as OXPHOS, and as a result, activates AMPK. The activation of AMPK inhibits mTORC1, as described previously in Reference [66].

#### 3.1.3. AMPK-Mediated Control of Metabolic Pathways

AMPK is at the junction of metabolic and genomic stress signals, controlling both growth factor activity and DNA damage response signaling. Upon activation, AMPK stimulates metabolic alterations to restrain energy expenditure (or increase energy production) and to make the necessary adjustments in response to specific metabolic stress. AMPK activation causes nutrient mobilization and catabolism for mitochondrial ATP production to restore energy homeostasis, increases glucose uptake and fatty acid oxidation in muscles, and upregulates the expression of various metabolic genes (e.g., GLUT4, uncoupling protein 3, and cytochrome c) [68]. Consequently, AMPK is considered a sensor/modulator of intermediary metabolism that directs cellular events to increase energy availability and sustain high-energy phosphate levels. AMPK is also a central mediator of liver kinase B1, which inhibits mTOR. Moreover, AMPK is necessary for cell cycle arrest at G1 under conditions of limited nutrient supply as it phosphorylates the tumor suppressors p53 and p27 [69,70].

Extensive data support that AMPK inhibits essentially all anabolic pathways that promote cell growth, such as those that control fatty acid, phospholipid, protein, and ribosomal RNA synthesis [68]. Therefore, it is not surprising that AMPK might hinder cancer cell growth, given that a hallmark of tumor cells is their ability to rapidly grow and divide, which requires a tremendous amount of energy. However, AMPK also inhibits the central steps of protein metabolism in muscle cells, contributing to the development of muscle wasting, and can induce the expression of atrogenes in skeletal muscle [71]. Further investigation into the role of AMPK in muscle wasting-related disturbances of protein turnover is warranted.

Collectively, this evidence indicates that muscle wasting is a manifestation of the sickness behavior correlated to the mechanisms of tolerance.

### 3.2. Cancer-Related Inflammation and Muscle Wasting

Cancer-related inflammation is intimately linked to the pathogenesis of the changes in energy metabolism seen in cachectic cancer patients [72]. In advanced cancer patients with the overt cancer-cachexia syndrome, the immunological function is severely impaired, characterized by a deficit in cell-mediated immunity associated with chronic inflammation and high levels of proinflammatory cytokines (IL-1, IL-6, and TNF-α) and acute-phase inflammatory proteins [fibrinogen and C-reactive protein (CRP)]. The synthesis of acute-phase proteins is upregulated at the expense of the production of other proteins, which are relevant for muscle anabolism, such as albumin and transferrin, whose production is decreased [73,74].

These proinflammatory cytokines have been implicated in the pathogenesis of metabolic disruptions and symptoms associated with cancer cachexia [75]. Not less important in inducing metabolic reprogramming is the hypoxic microenvironment where the cancer cells and immune cells live and interact [22]. In particular, macrophages, which are the key mediators of innate immunity and play a role in the orchestration of specific immunity, have been shown to exert a central role in these events. M1 macrophages play a role in secreting proinflammatory cytokines and directly inducing, metabolic changes that accompany the cachectic syndrome. Indeed, proinflammatory cytokines (such as IL-6, IL-1, TNF-α) can directly influence energy metabolism by modulating glucose metabolism, regulating the function of LPL [76], and by increasing protein degradation, which causes a subsequent decrease in lean body mass [31].

#### 3.2.1. IL-6 and STAT3 Crosstalk in Muscle Wasting

Mediators of inflammation act via the signal transducer and activator of transcription 3 (STAT3) [77], a master regulator of cell metabolism that regulates glucose metabolism, thereby favoring the less efficient process of glucose fermentation through aerobic glycolysis for energy production over mitochondrial OXPHOS [78]. During cancer evolution, muscle metabolism is subjected to extensive pathophysiologic regulation, and the imbalance between protein synthesis and degradation leads to muscle protein loss [79]. Findings from preclinical studies suggest that systemic inflammation leads to cachexia-related muscle wasting through STAT3/NF-κB signaling and consequent ubiquitin-proteasome system-mediated proteolysis [80]. Clinically, systemic inflammation in lung cancer patients correlates with the local inflammatory response in skeletal muscle mediated by elevated NF-κB activity [67]. Moreover, inhibiting STAT3 counteracts muscle wasting in cachectic tumor-bearing mice by inhibiting protein breakdown [81].

Experimental studies have implicated IL-6 as the main factor in the metabolic changes associated with cancer-related cachexia; IL-6 is also, at least to some extent, responsible for inflammation-related muscle wasting [80]. Increased muscle wasting was noted in murine models following high-dose or long-term treatment with IL-6 [82]. Inhibiting IL-6 signaling by prolonged administration of an IL-6 receptor (IL-6R) antibody prevented the muscle alterations observed in IL-6 transgenic mice, suggesting that it has a permissive role in the progression of skeletal muscle wasting and subsequent weight loss. The IL-6 activity on muscle mass is dose- and time-dependent. Specifically, no pro-atrophy role for IL-6 has been demonstrated experimentally with short-term and/or low-dose treatment, whereas continuous and systemic IL-6 exposure induces catabolic effects and muscle wasting [83].

IL-6 binds ligand-specific α-receptors (IL-6Rα, also known as gp80) in either the membrane-bound or soluble form to activate signaling [84]. This ligand–receptor complex binds the ubiquitously expressed type I cytokine receptor IL-6 signal transducer (gp130) to activate three main pathways: the STAT1/3 pathway, extracellular signal-regulated kinase (ERK) pathway, and PI3K/Akt/mTOR pathways. Here, IL-6 binding to gp130 activates the associated JAKs, which then engage SH2-containing proteins, including STAT3. Phosphorylation of STAT3 on tyrosine 705 leads to dimerization, nuclear translocation, DNA binding, and target gene regulation. Signaling can be blocked at the level of JAK or STAT3 by feedback inhibition of STAT3 target genes, including suppressors of cytokine signaling (SOCS), mainly SOCS3 [85]. Further, STAT3 is dephosphorylated by T-cell protein tyrosine phosphatase/protein tyrosine phosphatase and nonreceptor type 2 (TC-PTP/PTPN2) in the nucleus and cytoplasm [86].

All the pathways induced by IL-6 signaling, i.e., ERK, PI3K/Akt/mTOR, and STAT-3 pathway have been associated with muscle wasting [87,88]; however, the Janus kinase (JAK)/STAT is the most well-studied pathway.

In experimental models, mice inoculated with Chinese hamster ovary (CHO) cells expressing recombinant human IL-6 showed considerable wasting, with significant weight loss and proportionally greater muscle mass depletion, compared with control mice, which maintained their tumor-free body mass throughout the experiment [81]. Consistent with specific IL-6 signaling in muscle, STAT3 expression was significantly higher in all muscles in the mice inoculated with IL-6-expressing CHO cells. Furthermore, the muscle wasting progressed with prolonged IL-6 administration and the RNA expression of STAT3 and its target genes SOCS3, fibrinogen, and LBP1 were concomitantly elevated [81]. Pump-based administration of recombinant murine IL-6 for 1 week also caused weight loss, muscle wasting, and increased STAT3 levels in skeletal muscles. Thus, IL-6 is sufficient to activate muscle STAT3 and trigger wasting [81]. Conversely, local inhibition of STAT3 weakens or counteracts muscle wasting in response to IL-6 administration, revealing that STAT3 activation is primarily responsible for muscle wasting downstream of IL-6.

Another mechanism by which IL-6 contributes to muscle wasting is via blocking insulin synthesis by pancreatic beta cells. This blockade induces insulin resistance and impairs insulin signaling [89]. Moreover, IL-6 induces TLR-4 gene expression via STAT-3, which is one of the main mechanisms underlying insulin resistance in human skeletal muscle [90]. As a result, glucose usage by skeletal muscle is impaired, and glucose is redirected to the liver, as insulin does not affect hepatic glucokinase, which differs from the hexokinase found in myocytes [91]. In this situation, oxidation of nonessential amino acids is required to meet the energy demands of muscles, resulting in a negative nitrogen balance. To satisfy these energy needs, IL-6 increases protein degradation, which further decreases lean body mass [31]. In addition, IL-6 interferes with the growth hormone/IGF-1 axis that mediates muscle anabolism [92].

As described, the IL-6/STAT3 signaling cascade intersects with the PI3K/Akt/mTOR pathway that modulates muscle protein synthesis by regulating glucose metabolism. Therefore, the PI3K/Akt/mTOR pathway plays a central role in inflammation-mediated muscle wasting. mTOR signaling was inhibited in muscle during cachexia progression in ApcMin/+ mice. This effect was accompanied by increased circulatory levels of IL-6 [93]. In vivo and in vitro studies have shown that IL-6-induced myotube atrophy correlates with increased STAT3 and AMPK phosphorylation and reduced mTOR signaling [94]. AMPK is a recognized mTOR inhibitor and a downstream target of IL-6 signaling. When cachexia progresses, muscle AMPK activity increases in ApcMin/− mice [93]. Interestingly, exercise can slow the progression of cachexia by inhibiting IL-6-induced AMPK activation [95]. Further elucidation of these pathways could contribute to the identification of potential targets for anti-cachexia treatments.

#### 3.2.2. IL-6, Cancer-Related Anemia, and Muscle Wasting

The central role played by IL-6 in inducing cancer-related anemia and its relationships with the incidence and severity of muscle wasting is noteworthy. Anemia associated with chronic inflammation and deficiency of functional iron is a typical feature of advanced neoplastic disease [96]. While it is rarely mentioned as a typical symptom of cachexia, it has an extremely important role in the etiopathogenesis of muscle wasting. Indeed, the development of anemia in conjunction with cachexia has been documented, and the presence of anemia is a biochemical marker that should be used to diagnose cachexia [97]. Recent work has shown that the presence of anemia significantly influences the rate of skeletal muscle loss: anemic patients lose significantly more muscle and at an accelerated rate, suggesting that they might be at an increased risk for muscle wasting and weight loss, and thus, may have worse survival outcomes [98]. Indeed, cancer-related anemia affects the ability of erythrocytes to efficiently deliver oxygen to peripheral tissues. Impaired oxygen delivery may exacerbate the rate of muscle loss by decreasing the ability of tissues to oxidize metabolic substrates. Furthermore, anemia in chronic diseased states is associated with functional iron deficiency, which has been linked to low iron levels and the consequent inhibition of heme synthesis [99]. Considering that heme is a fundamental component of myoglobin [100], cancer-related anemia potentially exacerbates the etiopathogenesis of muscle wasting.

Since cancer-related anemia is closely related to chronic inflammation, we believe that it should be included in the criteria to define the cancer cachexia syndrome as it strongly contributes to determining its metabolic characteristics. Therefore, treatment of cancer-related anemia cannot be excluded from a multitargeted treatment approach for cancer cachexia. In fact, the treatment of each cachexia symptom could be futile, if not accompanied by the correction of cancer-related anemia.

#### 3.2.3. IL-6, Leptin, and Muscle Wasting

Several research articles from our group have shown that IL-6 levels in advanced cancer patients correlate with leptin, the main marker of body composition and nutritional status [101,102]. In particular, in patients with advanced ovarian cancer, rising IL-6 levels were inversely related with a decreased BMI, body weight, and lean body mass, and were also associated with a progressive decrease in leptin levels with the lowest values observed near the time of death [1].

Leptin has pleiotropic functions, including the modulation of glucose and energy metabolism [103]. Some studies have demonstrated that both glucose and pyruvate stimulate leptin release by increasing its intracellular de novo synthesis, conversely, their lack inhibits leptin synthesis [104]. Leptinemia appears to tightly follow variations in plasma insulin and glucose concentrations, independent of changes in adipose tissue mass. Additionally, the conversion of pyruvate to acetyl CoA is a key regulator of de novo leptin biosynthesis [105]. Therefore, the fact that both the tumor cells and the activated immune cells prefer glycolysis over OXPHOS could be associated with the inhibition of leptin synthesis, which can be regarded as an indicator of the state of energy production.

Accordingly, the changes in energy metabolism induced by the chronic action of proinflammatory cytokines, mainly by IL-6, in cancer cachexia alter insulinemia and glucose utilization for energy. This, along with anorexia and energy deprivation, leads to reduced leptin transcription in adipose tissue and low plasma leptin levels [106].

Moreover, in muscle wasting and sarcopenia, low leptin levels reflect a state of persistent energy depletion and are directly related to reduced muscle mass and function [107].

## 4. Role of Chemo/Radiotherapy in Inducing Cachexia

Not less important in patients that develop/present cachexia as an expression of the tolerance phase, and in particular in those who develop resistance to antineoplastic treatments, is the role of chemo/radiotherapy in inducing cachexia, as suggested by some preclinical and clinical studies [108]. In this regard, as already discussed above, it should be firstly specified that tumor response to chemotherapy with the resolution of disease-related inflammation-mediated molecular and metabolic pathways involved in the pathogenesis of cachexia, should be associated with improvement of this syndrome. Consistently, Patel JD et al. [109] demonstrated in a population of 2301 advanced, non-squamous non-small-cell-lung-cancer patients (which are very often affected by cachexia at diagnosis), that tumor response rate, disease control, and PFS were significantly associated with weight gain during chemotherapy, thereby supporting the notion that improvement of cachexia during treatment may be an early indicator of clinical benefit.

As regards the pro-cachectic mechanisms of chemo-radiotherapy, some data showed that antineoplastic agents can contribute to sarcopenia and cachexia [110]. For example, platinum regimens have been associated with weight loss through the ability to induce pro-cachectic cytokines and myostatin [111]. Chemotherapy-induced cachexia is mainly mediated by NF-kB, ERK 1/2, p38 mitogen-activated protein kinases pathways, independently from the ubiquitin-proteasome system [112]. Chemotherapeutic treatment affects skeletal muscle in cancer patients, and also through effects on reactive oxygen species production and mitochondrial dysfunction [113]. Pin et al. [114] showed that chemotherapy may induce cachexia by specific alterations in energy metabolism, i.e., glucose, amino acids, and lipid metabolic pathways, different from those induced specifically by cancer cachexia and associated inflammation. Very recently, Amrute-Naya M, et al. [115] demonstrated that chemotherapeutic drugs disrupt sarcomere organization and thereby the contractile ability of skeletal muscle cells by inducing the disruption of the transcriptional activity of SETD7 histone methyltransferase and p300 histone acetyltransferase with consequent severe loss of the molecular motor protein MyHC-II.

## 5. Role of Inflammation, Oxidative Stress, and Energy Pathways-Related Biomarkers for Early Detection of Cancer Cachexia

The inflammatory response with alterations of energy metabolism associated with the pathogenesis of cachexia leads to changes of circulating levels of several related parameters in cancer patients. Several papers from our group demonstrated that serum levels of proinflammatory cytokines, and particularly IL-6, are positively associated with ROS levels and inversely with antioxidant enzymes in advanced cancer patients [102,116,117]. The inflammatory response is also inversely related to leptin levels that, together with IL-6, may be a useful prognostic marker of disease outcome as demonstrated by us in a population of advanced ovarian cancer [117]. Indeed, leptin is closely associated with cytokine levels, mainly IL-6, and has been identified as a mediator of the metabolic and immunological changes observed in advanced cancer patients [118]. Leptin changes closely reflected changes in IL-6, according to tumor objective response or progression [117]. It could thus be hypothesized that the changes observed with leptin parallel the changes in energy metabolism that are induced by cytokines, long before the metabolic changes induce a significant loss in body weight. Then, IL-6 and leptin could represent early markers of the main symptoms and metabolic alterations associated with the pathogenesis of cancer cachexia. The search for parameters useful to diagnose cachexia according to its degree, and to improve its recognition in the early stages to improve a more effective therapeutic approach, is a recognized need by researchers operating in this field [4]. In this regard, recently Argiles JM et al. [119] developed and validated a tool to assess the different stages of cachexia, which includes several important parameters of inflammation/metabolic disturbances/immunosuppression (i.e., CRP, IL-6, albumin, lactate, triglycerides, urea, hemoglobin, ROS, and glucose tolerance test/insulinemia). Their results showed that CASCO may represent a new valid tool for the quantitative staging of cachectic cancer patients [119].

## 6. Perspective for a Targeted Metabolic-Driven Therapeutic Approach for Cancer Cachexia

The above evidence indicates that muscle wasting during the evolution of neoplastic disease involves reduced protein synthesis and an associated increase in muscle degradation. Moreover, the increased proliferation rate of cancer cells and the chronic inflammatory response evoked by the defective immune response in the resistance phase are associated with increased energy expenditure, altered energy metabolism, and the concomitant onset of cancer-related anorexia. These events evoke energy stress with consequent protective inhibition of the PI3K/AKT/mTOR pathway and halted protein synthesis. Chronic inflammation induces proteolysis, particularly mediated by IL-6, with the release of amino acids. This effect is useful for supporting the increased energy demand through β-oxidation and is probably also an attempt to reactivate mTOR. Importantly, IL-6 is considered a mediator of cancer anorexia and the main inducer of cancer anemia, and both conditions eventually inhibit the mTOR axis, thereby establishing a vicious cycle that augments muscle wasting. Strategies to reverse this condition should consider two factors: counteracting anorexia and anemia, and inhibiting the activity of inflammatory cytokines, particularly IL-6.

These observations justify a multitargeted therapy approach for neoplastic cachexia, as we have emphasized in our previous publications, where specific modulators of inflammation, energy metabolism, and associated oxidative stress find their broad etiopathogenic rationale.

### 6.1. Drugs Targeting Inflammation

Approved drugs to treat cachexia include progestins (megestrol acetate and medroxyprogesterone acetate) for their ability to stimulate appetite and body weight demonstrated in several clinical trials [120]. Their mechanism of action is mediated mainly by their capacity to decrease the synthesis of proinflammatory cytokines, downregulate the ubiquitin-proteasome pathway, and stimulate the orexigenic neuropeptide Y [120]. However, these drugs do not seem to exert a significant impact on muscle mass, while they may have significant adverse effects, such as thromboembolic phenomena, peripheral edema, hyperglycemia, hypertension, adrenal suppression, and adrenal insufficiency. Therefore, their use should warrant a careful evaluation of their potential toxicities especially in hospitalized and elderly patients [121,122].

Among anti-inflammatory agents, a COX-2 inhibitor, which has been tested alone [123,124] and in combination regimens for cancer cachexia [125,126], should be able to improve several features of cancer cachexia including loss of body weight, muscle strength, and lean body mass in parallel with a decrease in circulating levels of pro-inflammatory cytokines, particularly IL-6 and CRP. Notably, these compounds exert their anticachectic effect through their ability to downregulate the proinflammatory cascade (prostaglandin, TNF, IL-6) and the Cox-2-mediated activation of proteolytic pathways, as well as inhibiting the GH-IGF1-mediated protein anabolism [127]. Notable, in experimental models associated with muscle wasting, celecoxib was able to prevent the rise in blood levels of lactate, the inhibition of the peripheral response to insulin and hepatic glycolysis, and tended to attenuate the decrease in food intake [128].

Specific therapeutic targets against proinflammatory cytokines (IL-6, TNFα, IL-1, etc.) have been proposed for testing in clinical trials, based on the preclinical investigation. However, none of these drugs has been yet approved for the indication of cancer cachexia [2]. IL-6 represents the main target cytokine for the treatment of muscle wasting and a preclinical animal study with the IL-6 inhibitor tocilizumab showed promising results [129]. Additionally, in preliminary clinical trials in patients with lung cancer and cachexia, the humanized anti-IL-6 antibody tocilizumab is safe and effective against cachexia-related symptoms [130,131]. Interestingly, considering the mediating role of the JAK/STAT3 pathway in IL-6-induced muscle wasting, the role of the recently developed JAK and STAT3 inhibitors (i.e., ruxolitinib) on muscle mass and function should be further investigated especially when considering the significant benefit obtained specifically in ameliorating the cachectic symptoms in a large clinical trial in patients with multiple myeloma [132].

Among nutraceuticals, curcumin also proved to be useful as an anticachectic agent [133,134]. Its efficacy may be mainly due to the ability to inhibit STAT-3-induced NF-kb signaling and increase sirtuin-1 activity, thereby counteracting muscle proteolysis and wasting [135]. 

Additionally, lactoferrin can lessen inflammation associated with M1 macrophage polarization [136] and positively modulate the related changes in iron metabolism (iron trafficking and storage), mobilizing iron from deposits and increasing its availability for erythropoiesis [137].

### 6.2. Drugs Targeting Oxidative Stress

Additionally, antioxidants such as glutathione, N-acetyl cysteine, and quercetin, can revert cancer-induced muscle wasting by reducing both oxidative stress and inflammation by modulating the ROS-mediated activation of the inflammasome pathway. Indeed, ROS may affect the protein synthesis and degradation involved in muscle wasting and they are a known modulator of the PI3K/Akt/mTORC1 pathway, calpain and ubiquitin-proteasome mediated proteolysis, and autophagic degradation of muscle mass [138]. Moreover, in an animal experimental model, ROS-induced alterations affected muscle quality and function [139].

### 6.3. Modulators of Energy Metabolic Pathways

L-carnitine was also shown to effectively improve lean body mass in cachectic cancer patients, most likely for its ability to increase oxidative mitochondrial energy metabolism and exert antioxidant effects. In a randomized double-blinded trial, 72 patients with advanced pancreatic adenocarcinoma received 4 g carnitine or placebo for 12 weeks, and the treatment group achieved an improvement in body cell mass [140]. Another study by our group showed that treatment with l-carnitine (6 g/day) in a population of 12 patients with different cancer types at advanced stage led to an increase in lean mass and appetite associated with a decrease in fatigue and oxidative stress over 4 weeks of treatment [141]. The metabolic role of l-carnitine as an anticachectic agent is likely related to its ability to modulate the carnitine/carnitine palmitoyl transferase system. This is the rate-limiting step of fatty acid oxidation/ketogenesis, which is activated in catabolic states such as cachexia, in association with gluconeogenesis, and decreased lipogenesis [142]. In experimental preclinical models, l-carnitine was shown to ameliorate cancer cachexia and increase muscle mass by increasing the activities of carnitine palmitoyl transferase I and II [143,144]. Moreover, l-carnitine can modulate peroxisome proliferator-activated receptor gamma-1 alpha, a master regulator of metabolism, which induces hepatic gluconeogenesis and fatty acid oxidation in the catabolic state [143].

Among nutritional supplements, amino acids have been tested as a targeted therapy of cancer cachexia for their critical role in promoting protein synthesis in muscle tissue. The emerging role of some amino acids, in particular branched-chain amino acids (BCAAs), in reversing the anabolic muscle resistance is being reported [145]. Noteworthy, hydroxyl-methyl butyrate (HMB) can up-regulate the phosphorylation of mTOR and downregulate the Akt/FoxO and MuRF1 which controls autophagy and ubiquitin-dependent proteolytic systems [127]. Accordingly, amino acids are demonstrated to ameliorate muscle loss in cancer cachexia [146]. Interestingly, in advanced cancer patients, HMB combined with other amino acids in supplements resulted in a significant gain in lean body mass as demonstrated by randomized clinical trials [147,148] and a systematic review [149].

## 7. Discussion and Conclusions

The implementation of the knowledge of the early mechanisms involved in the onset of cancer cachexia and the development of its complex metabolic alterations may certainly improve a timely diagnosis and a more adequate treatment aimed to specifically target its pathogenetic mechanisms [150].

It is our opinion that, as explained in the present review, cancer cachexia should be considered the evidence of symptoms related to tolerance, that is resulting from the failure of resistance, and it should be considered as the final attempt by the body to counteract cancer growth. In light of these notions, the understanding of cachexia opens important theological questions about the relevance of the relationship between the tumor and the patient’s body that hosts it, and therefore, about the relationships between mind and cancer. This highlights once again, that the tumor is not extraneous but the final evidence of the dysregulation of an entire organism where the death of the subject coincides with the death of the neoplasm that caused it.

Therefore, a better knowledge of the mechanisms of resistance and tolerance discussed in the present review as crucial events involved in the pathogenesis and development of cancer cachexia surely could help clinicians to be aware of the relevance of cachexia incidence in the evolution of cancer disease and improve its early recognition. Consistently, with more effective treatment with agents targeting the inflammatory pathways, the related changes in the oxido-reductive status, and the relevant energy metabolism derangements could be early started and offer a greater advantage in the prevention and treatment of this syndrome.

There is no doubt that anticachectic therapies have an irreplaceable role in cases of reversible cachexia where, if harmoniously associated with effective antineoplastic therapies, they can effectively contribute to maintain a quality of life and promote the efficacy of the treatment. Meanwhile, the role of these therapies is very different in the different stages of irreversible cachexia that leads to death, where anticachectic therapy can be useful only to improve the quality of life. Allowing the patient and his family to get a better awareness of the final stages of life, thereby opening to the best spiritual remodulation of the final event, death. Thus, in this context, the increase in the number of days obtained with the use of these therapies certainly has its clinical significance but its spiritual role must also emerge.
